# The transcriptional activity of hepatocyte nuclear factor 4 alpha is inhibited *via* phosphorylation by ERK1/2

**DOI:** 10.1371/journal.pone.0172020

**Published:** 2017-02-14

**Authors:** Borbála Vető, Dóra Bojcsuk, Caroline Bacquet, Judit Kiss, Szabolcs Sipeki, Ludovic Martin, László Buday, Bálint L. Bálint, Tamás Arányi

**Affiliations:** 1 Institute of Enzymology, Research Center for Natural Sciences, Hungarian Academy of Sciences, Budapest, Hungary; 2 Doctoral School of Molecular Medicine, Semmelweis University, Budapest, Hungary; 3 Genomic Medicine and Bioinformatic Core Facility, Department of Biochemistry and Molecular Biology, Medical Faculty, University of Debrecen, Debrecen, Hungary; 4 Department of Medical Chemistry, Molecular Biology and Pathobiochemistry, Semmelweis University, Budapest, Hungary; 5 CNRS UMR 6214, INSERM U1083, University of Angers, Angers, France; Institute of Genetics and Molecular and Cellular Biology, FRANCE

## Abstract

Hepatocyte nuclear factor 4 alpha (HNF4α) nuclear receptor is a master regulator of hepatocyte development, nutrient transport and metabolism. HNF4α is regulated both at the transcriptional and post-transcriptional levels by different mechanisms. Several kinases (PKA, PKC, AMPK) were shown to phosphorylate and decrease the activity of HNF4α. Activation of the ERK1/2 signalling pathway, inducing proliferation and survival, inhibits the expression of HNF4α. However, based on our previous results we hypothesized that HNF4α is also regulated at the post-transcriptional level by ERK1/2. Here we show that ERK1/2 is capable of directly phosphorylating HNF4α *in vitro* at several phosphorylation sites including residues previously shown to be targeted by other kinases, as well. Furthermore, we also demonstrate that phosphorylation of HNF4α leads to a reduced *trans*-activational capacity of the nuclear receptor in luciferase reporter gene assay. We confirm the functional relevance of these findings by demonstrating with ChIP-qPCR experiments that 30-minute activation of ERK1/2 leads to reduced chromatin binding of HNF4α. Accordingly, we have observed decreasing but not disappearing binding of HNF4α to the target genes. In addition, 24-hour activation of the pathway further decreased HNF4α chromatin binding to specific loci in ChIP-qPCR experiments, which confirms the previous reports on the decreased expression of the *HNF4a* gene due to ERK1/2 activation. Our data suggest that the ERK1/2 pathway plays an important role in the regulation of HNF4α-dependent hepatic gene expression.

## Introduction

Hepatocyte nuclear factor 4α (HNF4α) is a protein that was first identified as an activator of the *Transthyretin* gene expressed upon binding to a *cis*-regulatory element [1]. The transcription factor (TF) was then purified [2], and the analysis of the gene showed that it is a member of the nuclear receptor superfamily. Later studies demonstrated its fundamental role in several processes [3]. Accordingly, the HNF4α knockout mouse model is embryonic lethal since the TF is required for gastrulation and liver development [4].

In adults, HNF4α is expressed in the liver, pancreas, intestines and the kidney [5]. It participates in the regulation of a multitude of genes, in addition, it is considered as a master regulator in hepatocytes [6]. Genes of lipid and glucose metabolism [7], transporters and transcription factors are among the most important targets of the HNF4α protein [8, 9]. Although it reacts to environmental stresses, such as fasting or feeding, it has long been considered as an orphan receptor [10].

Crystallization studies have shown, however, that it can be co-crystallized as a homodimer with long-chain fatty acids in its ligand-binding pocket [11]. Furthermore, the ligand seemed to be a constitutive activator, suggesting that HNF4α is constitutively active [12]. Since several functional studies have demonstrated that the transcription factor is not constitutively activating its target genes, the investigation of post-translational modifications and the search for interacting partners has begun [13, 14]. These latter experiments revealed the interaction of HNF4α with different transcription factors (e.g. farnesoid X receptor FXR) [15], co-activators and co-repressors [15, 16].

The study of potential post-translational modifications has identified several acetylation, ubiquitilation and phosphorylation sites. Mass spectrometry analyses of post-translational modifications by phosphopeptide mapping showed that HNF4α can be phosphorylated in the hepatoma-derived HepG2 cell line at several sites [13]. Different kinase cascades were also shown to phosphorylate the protein at specific residues. Protein kinase C (PKC) has the most important inhibitory effect by phosphorylating serine 78 [17]. Protein kinase A (PKA) [18] and p38 [19] were also shown to phosphorylate the protein. However, these latter results seem to be controversial. Finally, AMP activated kinase (AMPK) targets serine 313 [20] and the phosphorylation of this amino acid residue was also shown to decrease the activity of the transcription factor, however, to a lesser extent than PKC [17].

*ABCC6* is a gene of the ATP-binding cassette (ABC) transporter family. It encodes a protein mainly expressed in the basolateral membrane of hepatocytes, which transports an unknown substrate from the cells to the bloodstream. Loss-of-function recessive mutations of the gene lead to the development of ectopic soft tissue calcification and the fragmentation of elastic fibres. The resulting syndrome is called *Pseudoxanthoma elasticum* (PXE), characterized by dermatologic, ocular and cardiovascular symptoms (reviewed in [21]).

We have identified several *cis*-regulatory elements at the gene promoter and a primate-specific sequence in the first intron of the gene [22]. We have also shown that a transcription factor network including HNF4α, CCAAT/enhancer binding protein (CeBP) α and β bind these *cis*-regulatory elements [23]. Our results clearly suggested that HNF4α orchestrates this network and it is responsible for the tissue- and cell-type specific expression of *ABCC6* [22, 24].

While investigating the transcriptional regulation of the human *ABCC6* gene in human cell lines, we observed that the activation of several kinase cascades (PKC, AMPK and ERK1/2) inhibits the expression of the gene *via* HNF4α [24]. In our model system, HNF4α was introduced in a eukaryotic expression plasmid and the gene expression was not sensitive to ERK1/2. Therefore, in the present study we investigated whether HNF4α is a direct target of ERK1/2 phosphorylation. Here we show that ERK1/2 directly phosphorylates HNF4α, which decreases the *trans*-activating capacity of the transcription factor.

## Results

We have shown that the expression of *ABCC6* is inhibited by ERK1/2 kinase *via* HNF4α [24]. It has also been reported that ERK1/2 can decrease HNF4α expression [25]. However, in our experimental system we did not investigate the effect of ERK1/2 on the endogenous *HNF4a* gene, but we rather used a plasmid-based eukaryotic expression system, which was not sensitive to ERK1/2. Thus, we hypothesized that ERK1/2 directly inhibits the HNF4α protein.

### HNF4α is phosphorylated by ERK1/2

**Fig 1:**
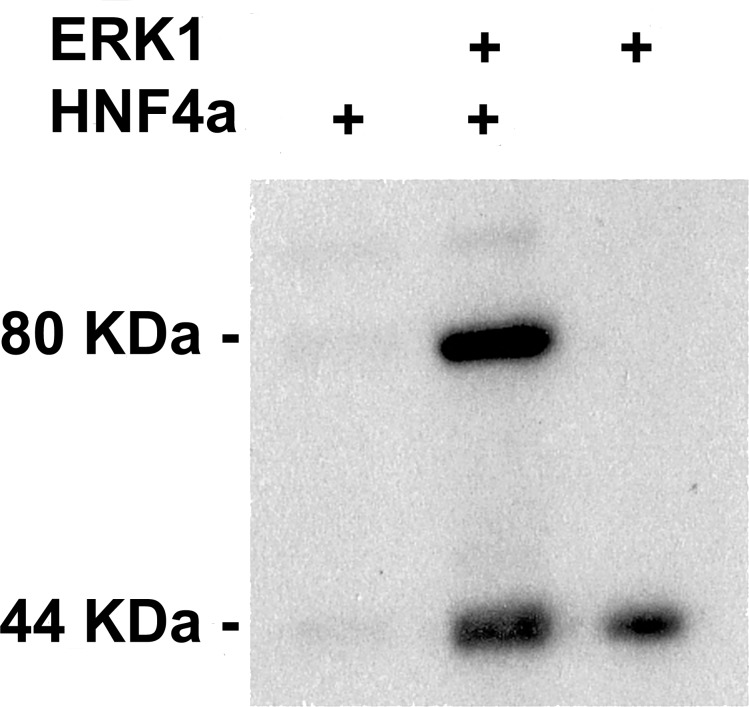
*In vitro* phosphorylation assay of HNF4α by ERK1 kinase. The phosphorylation assay was carried out with *in vitro* translated human recombinant HNF4α protein, ERK1 kinase and radioactively labelled [γ-^32^P] ATP. HNF4α does not contain any post-translational modifications, but it has a GST-tag at N-terminal. The samples were run on SDS-PAGE and subjected to autoradiography. ERK1 kinase is capable of autophosphorylation.

In order to confirm this hypothesis, we carried out *in vitro* phosphorylation assay on HNF4α by ERK1. We used *in vitro* translated human recombinant HNF4α protein—not decorated with any post-translational modifications–in fusion with GST-tag at N-terminal, ERK1 kinase and radioactively labelled [γ-^32^P] ATP. The samples were run on SDS-PAGE and subjected to autoradiography. As shown on **Fig 1**, ERK1 kinase is capable of autophosphorylation, [26], but it also phosphorylated HNF4α, as indicated by a band in the middle lane.

### Phosphopeptide mapping of detected phosphorylation sites

**Fig 2:**
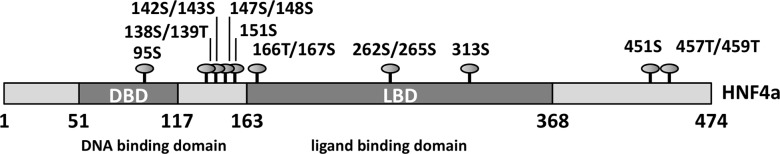
Phosphorylated sites on HNF4α protein by ERK1 kinase detected by mass spectrometry. Non-radioactively phosphorylated HNF4α by ERK1/2 was cut out from the polyacrilamid gel. Tryptic peptide fragments were analysed by mass spectrometry. Affected phosphorylation sites (indicated by lollipops) appear in the DNA binding domain (DBD), the hinge, the ligand-binding domain (LBD) and the C-terminus of HNF4α, as well. The phosphorylated amino acid residues can be attributed to either previously described sites (S138/T139, S142/S143, S147/S148, S151, T166/S167, S313) or new positions reported first here (S95, S262/S265, S451, T457/T459).

Subsequently, we asked which serine/threonine residue(s) are phosphorylated. For this, the above described phosphorylation assay was performed in duplicates, where one of the samples was labelled only with non-radioactive phosphate. This ERK1-phosphorylated HNF4α sample was cut out from the gel and subjected to mass spectrometry analysis. We have identified a great number of phosphorylated amino acid residues, although, if two sites were adjacent, the method we used could not distinguish between them (**Fig 2** and **Table 1**). Affected phosphorylation sites appear in the DNA binding domain, the hinge, the ligand-binding domain and the C-terminus of HNF4α, as well. In spite of the numerous positions identified, interestingly, the previously reported main phosphorylation site, the S87 residue of the human protein (corresponding to rat S78) has not been found in our experiment (see Discussion) [17]. We have concluded that ERK1/2 is capable of phosphorylating human HNF4α at multiple sites, which occur at previously described (S138/T139, S142/S143, S147/S148, S151, T166/S167, S313) and new positions reported here first (S95, S262/S265, S451, T457/T459). Moreover, our results show that the ERK1-targeted positions overlap with the phosphorylation sites of other kinases, for instance PKA, p38 and AMPK (see Introduction and Discussion).

**Table 1:** Phosphorylated amino acid residues identified by mass spectrometry. *In vitro* phosphorylated HNF4α was subjected to mass spectrometry analysis. The fragments containing the phosphorylated amino acid residues correspond to phosphorylation sites on the HNF4α protein. Detected phosphorylation sites (serine/threonine residues) in the cryptic fragments are marked bold and underlined. Affected phosphorylation sites appear in the DNA binding domain (DBD), the hinge, the ligand-binding domain (LBD) and the C-terminus of HNF4α, as well.

Sequence	Identified phosphorylation sites	Part of HNF4α
KNHMY**S**CR	S95	DBD
QNERDRI**ST**RR**SS**YED	S138/T139	hinge
QNERDRISTRR**SS**YED	S142/S143	hinge
STRRSSYED**SS**LPSINALLQ	S147/S148	hinge
STRRSSYEDSSLP**S**IN	S151	hinge
EVLSRQI**TS**PVSGIN	T166/S167	LBD
HCPELAEM**S**RV**S**IR	S262, S265	LBD
GKIKRLR**S**QVQVSLED	S313	C-terminus
**S**AIPQP**T**I**T**KQE	S451, T457, T549	C-terminus

### HNF4α phosphorylation via ERK1/2 inhibits ABCC6 transcriptional activity

This great number of amino acid residues targeted by ERK1 phosphorylation raised the question of the functional relevance of the observed phenomenon. In order to answer this question, several phosphorylation sites were selected and investigated in luciferase reporter gene assay.

Five phosphorylation sites were chosen for further examination. Some of them were already studied previously, while we also investigated residues detected for the first time in the previous experiment. Mutations were designed for the following serine or threonine phosphorylation sites creating phosphomimetic (glutamate or aspartate) or neutral (alanine) mutants: S87D, T166A/S167D, S313D, S451E, T457A/T459E and S451E/T457A/T459E triple mutant. If two phosphorylation sites were adjacent or in very close proximity, both were mutated.

Serine 87 mutation to aspartate was chosen because this site is undoubtedly a target for PKC phosphorylation, which abolishes HNF4α activity [17]. Thus, this site served as a positive control in our experiments. T166/S167 is a site, which was described to be phosphorylated by p38α or p38β MAP kinases [13, 19], therefore also suggesting an important role of these two adjacent sites. The logic in our mutational screen was similar to that observed in the literature, i.e. to change only one site into a phosphomimetic mutation and mutate the other to a neutral one, if there were two adjacent phosphorylation sites. Accordingly, T166 was mutated into a neutral amino acid (alanine), whereas S167 became a phosphomimetic mutant (aspartate). The site S313D is the target of phosphorylation by AMPK [14]. Finally, we newly identified the sites S451 and T457/T459. Therefore, we designed a neutral/phosphomimetic double mutant, in this case the phosphomimetic mutant being glutamate. Lastly, S451/T457/T459 were mutated in order to see the effect of a triple mutant, by including the former S451E mutant. Thus, we intended to examine if these C-terminal amino acids take part in transcriptional regulation when phosphorylated.

**Fig 3:**
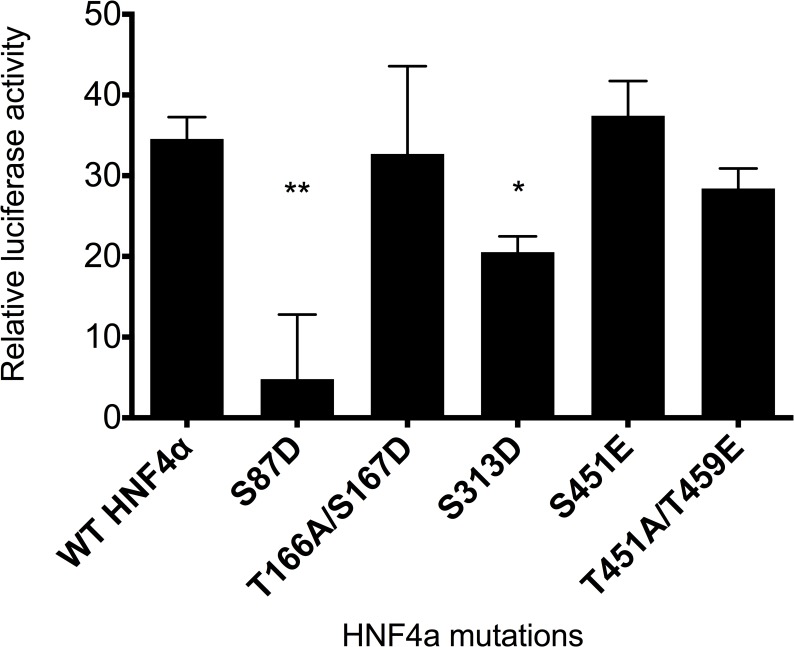
Luciferase assay measuring *ABCC6* promoter activity of HNF4α phosphomimetic mutants in HeLa cells. Mutations were designed for serine or threonine phosphorylation sites creating phosphomimetic (glutamate or aspartate) or neutral (alanine) mutants. For luciferase assays, triple co-transfection was performed with the phACCC6(-332/+72)Luc construct composed of the *ABCC6* promoter fragment, pcDNA5-FRT/TO plasmid encoding HNF4α variants and pRL-TK Renilla luciferase Control Reporter Vector. Luciferase activity was measured 48 hours after transfection. Relative luciferase activity was calculated by normalizing for background noise and for transfection efficiency by the co-transfected control reporter vector. The error bars represent S.D. Tukey-HSD test was performed. Significance versus WT HNF4 alpha is indicated by asterisks: *p<0.05; **p<0.01.

After creating all the mutants, co-transfection was performed with a luciferase reporter gene under the control of *ABCC6* promoter and different HNF4α mutants into HeLa cells lacking endogenous HNF4α expression. The obtained results were normalized first for the background noise, then for transfection efficiency by the co-transfected control reporter vector, which essentially represents transfection efficiency. As shown on **Fig 3**, wild type HNF4α results in similar activity to T166A/S167D, S451E and T457A/T459E, and S451E/T457A/T459E (data not shown), therefore, we regard these residues as not being responsible for implementing the effect of phosphorylation on transcriptional activity or gene expression in this assay. However, both S87D (used as positive control) and S313D have significant inhibitory effect on *ABCC6* promoter activity: they decrease the activity to approximately 15% and 55%, respectively, compared to control. In conclusion, the phosphorylation site S313 targeted by both ERK1 and AMPK is responsible to have an inhibitory effect on transcription.

### HNF4α and H3K27ac histone mark overlap in ChIP-Seq

In order to further confirm the functional relevance of HNF4α phosphorylation by ERK1/2, we carried out a cell-based assay, which reflects appropriately the endogenous intracellular processes. First, we screened control HepG2 cells for active HNF4α binding sites prior to selecting some genomic target loci to investigate the effect of ERK1/2 on HNF4α binding.

To detect active HNF4α binding sites in the HepG2 cells at the genome-wide level, we carried out chromatin immunoprecipitation followed by sequencing (ChIP-seq) with an antibody against HNF4α and—in a parallel experiment on chromatin prepared from the same HepG2 sample—with an antibody against acetylated histone 3 lysine 27 (H3K27ac). The H3K27ac covalent modification of the chromatin indicates active regulatory regions, which are often enhancers.

**Fig 4:**
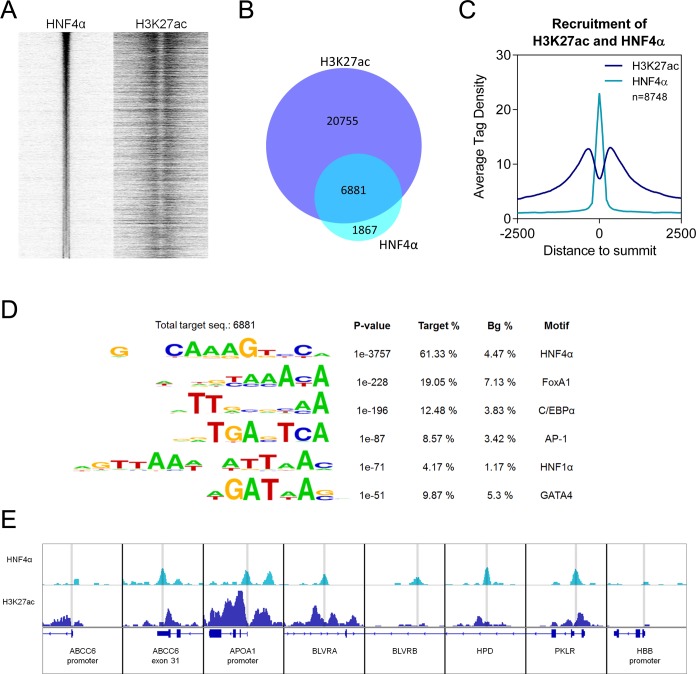
(**A**) Read distribution plot of HNF4α and H3K27ac upon vehicle treatment, relative to the 8,748 HNF4α peaks in 2-kb frames. Peaks are sorted according to HNF4α tag density. (**B**) Area-proportional Venn diagram illustrating the overlap between H3K27ac regions and HNF4α peaks. (**C**) Histogram shows the average tag density of vehicle-treated HNF4α peaks and H3K27ac signals at the sites of 8,748 HNF4α binding sites. (**D**) Motif enrichment of 8,748 HNF4α peaks. The P value and target and background (Bg) percentages are included for each motif. (**E**) IGV snapshot of HNF4α and H3K27ac ChIP-seq coverage representing eight selected genomic regions upon vehicle treatment. HBB promoter: negative control region. The interval scale is 50 in both cases. Peaks, highlighted with grey lines, represent the sites of the investigated HNF4α-target genomic regions.

From the ChIP-seq sample immunoprecipitated with HNF4α antibody, 8,748 transcription factor binding sites (TFBSs) could be identified (**S1 Table**). We have plotted these sites on a read distribution plot and we have sorted them based on tag density. Then we have matched the signal of the active histone mark H3K27ac (**Fig 4A**). **Fig 4B** illustrates on a Venn diagram that over 75% of HNF4α peaks overlap with H3K27ac peaks.

We have also plotted the average tag density of the HNF4α peaks and the H3K27ac signals, indicating that the HNF4α TFBSs represent a Gaussian distribution, whereas histone signals show the peak-valley-peak shape (**Fig 4C**). We have also observed that 451 peaks overlap with the TSS (+1 is part of the binding site) and in these cases, the average tag density of H3K27ac is twice as high as in the case of the distribution centered to HNF4 alpha peaks (**Fig 4C** and **S1 Fig**). Furthermore, 33% and 71% of the HNF4 alpha peaks are within +/- 5 kb and +/- 30 kb from the TSS, respectively. These results indicate that the predicted TFBSs follow the expected typical patterns and confirm that these are real TFBSs without significant non-specific binding.

To further validate the predicted TFBSs, we applied motif enrichment analysis on these regions. As expected, the consensus motif of HNF4α (61.33%) and its cooperative factors, such as forkhead box A1 (FoxA1) and C/EBPα, are enriched. Motif of the activator protein 1 (AP-1), HNF1 homeobox A (HNF1α) and GATA binding protein 4 (GATA4) were also identified (**Fig 4D**). By annotating the 8,748 HNF4α TFBSs, many of them are located near genes related to PPAR and insulin signalling or fatty acid metabolism. Furthermore, when we verified the pathways enriched for genes with HNF4α binding sites, the membrane transport-related ABC-transporter genes also appeared (**Table 2**). Therefore, we selected the following target genes for further experiments: *4-hydroxyphenylpyruvate dioxygenase* (*HPD*), *Pyruvate kinase*, *liver and red blood cell* (*PKLR*). We have also selected the *ABCC6* gene since we and others have reported that HNF4α binds the promoter of this gene [24, 27]. *Apolipoprotein A1* (*APOA1*) is a well-known target for HNF4α, therefore we also included it in the investigation. Indeed, our ChIP-seq results also showed that it is bound by HNF4α together with the presence of the H3K27ac, suggesting that they have active regulatory regions in HepG2 cells. Moreover, it is listed as a gene having a role in PPAR signalling in the KEGG pathways obtained from our ChIP-seq data. Finally, we have identified *Biliverdin A* (*BLVRA*) and *Biliverdin B* (*BLVRB*), which are closely connected to heme oxygenase in heme catabolism, a known target of HNF4α, which plays a role in anti-oxidative and anti-inflammatory defence mechanisms. We have also selected a negative control region: the β*-globin* promoter, which was devoid of acetylation and HNF4α binding (**Fig 4E**).

**Table 2:** Top 15 biological pathways related to the 8,748 HNF4α binding sites. Data derived from KEGG database.

Top 15 pathway terms	log P-value
Peroxisome	-11.59335105
Adherens junction	-10.88593944
Focal adhesion	-9.508833554
PPAR signaling pathway	-9.255948133
Leukocyte transendothelial migration	-9.21522799
Insulin signaling pathway	-9.164553569
Adipocytokine signaling pathway	-9.018794995
Glycine, serine and threonine metabolism	-8.616929048
Complement and coagulation cascades	-8.079259745
Axon guidance	-7.860829255
Primary bile acid biosynthesis	-7.152608222
Tight junction	-6.376981421
ErbB signaling pathway	-6.124131582
**ABC transporters**	**-5.556926349**
Fatty acid metabolism	-5.556926349

### ERK1/2 activation leads to reduced binding of HNF4α to specific genomic regions

In the following experiment we used the selected genomic target regions to investigate the dynamics of HNF4α binding to our genes of interest upon ERK1/2 induction in HepG2 cells. Since phosphorylation of proteins is a fast process, and happens within minutes [28], we decided to perform short-term (30 minutes) treatment of HepG2 cells with epidermal growth factor (EGF), which activates the ERK1/2 signalling cascade after binding to its receptor.

**Fig 5:**
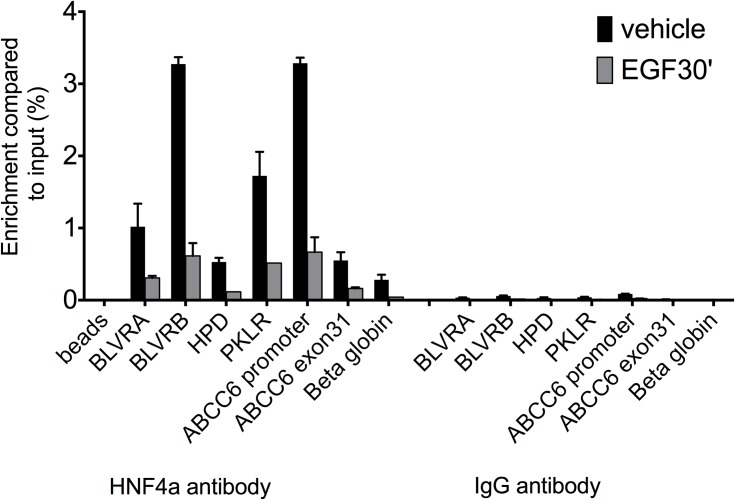
ChIP-qPCR results showing HNF4α occupancy on seven genomic regions of hepatic HNF4α target genes. Immunoprecipitation of chromatin from HepG2 cells was performed with anti-HNF4α or IgG antibody untreated or treated with EGF for 30 mins. Enrichment was compared to % input (y axis). HNF4α binding (black columns) is decreased after short EGF treatment (grey). BLVR A: Biliverdin A, BLVR B: Biliverdin B, HPD: 4-hydroxyphenylpyruvate dioxygenase, PKLR: Pyruvate kinase, liver and RBC, ABCC6: ATP-binding cassette subfamily C, member 6 and APOA1: Apolipoprotein A1. Beta globin: negative control region. Beads represent immunoprecipitation performed without any antibody. S.D. is indicated on the figure. Results of a representative experiment (n = 7) are shown.

In our ChIP-qPCR experiments, enrichments of immunoprecipitated target fragments by anti-HNF4α antibody were compared to the input fraction or to the selected negative control region (β-globin) for TF occupancy, where no binding of the protein of interest is expected. Similarly, we systematically performed another negative control, immunoprecipitation with IgG, used as non-specific antibody. We carried out 7 independent experiments under the same conditions in HepG2 cells and as hypothesized, 30 minutes treatment led to reduced HNF4α binding to the selected target sites, as shown on **Fig 5** and **S2 Fig.** We evaluated the effect of short-term EGF treatment by performing a one-sample t-test after normalization of the immunoprecipitated fraction from the treated cells for each target gene to their respective controls. Accordingly, highly significant effect was observed after short treatment (p<0.004). More precisely, after 30 minutes treatment, based on our ChIP-qPCR data, there is a substantial loss of HNF4α binding compared to the control case for the individual target genes, shown on **S2 Table**. This effect was further enhanced by 24h EGF treatment. Collectively, our data suggest that ERK1/2 phosphorylates HNF4α both *in vitro* and *in vivo*, leading to the reduced DNA binding capacity of the transcription factor at target loci.

## Discussion

We have shown previously that HNF4α regulates *ABCC6* gene expression. We have also demonstrated that ERK1/2 activation inhibits HNF4α-dependent *ABCC6* expression. We hypothesized that HNF4α is phosphorylated by ERK1/2 leading to its reduced *trans*-activational capacity in addition to the reduced expression of the *HNF4a* gene. Here we have demonstrated that ERK1 is able to phosphorylate HNF4α at several serine and threonine residues. We have also shown that phosphorylation of HNF4α inhibits its *trans*-activational capacity in reporter gene assay and its chromatin binding activity as determined by ChIP-qPCR confirming the physiological relevance of our findings.

HNF4α plays a major role in hepatic development and it is a master gene regulator in differentiated hepatocytes. It regulates thousands of genes playing important roles in glucose, lipid and amino acid metabolism, bile acid synthesis, detoxification and inflammation. Our results are in harmony with these findings. In our ChIP-seq experiments, in non-treated HepG2 cells we found almost 9000 genomic HNF4α binding sites associated with 5500 genes. More than 60% of them are canonical HNF4α elements, but binding motifs for FoxA1, C/EBPα and HNF1α were also enriched in the immunoprecipitated DNA fraction. These sequences are known to be bound by TFs interacting with HNF4α, thereby strengthening the validity of our findings. CEBPα has been previously reported as being part of a complex with HNF4α forming together an intricate regulatory network of hepatocyte gene expression [22, 29]. HNF1α is a transcription factor known to interact with HNF4α, where HNF4α enhances HNF1α-mediated activation of hepatic transcription [30]. Our data also indicated that we identified actively transcribed HNF4α-regulated genes since those sites were also occupied by the H3K27ac histone mark, a hallmark of active genes [31] (**Fig 4B**). Finally, we were able to identify typical HNF4α target genes in the immunoprecipitated fraction. Furthermore, the KEGG pathway analysis of all identified HNF4α target genes revealed similar cascades to those available as literature data. Among the typical genes *ABCC6*, *ABCA1*, *ALDOB (aldolase B)*, *APOA1*, *APOB*, *APOCIII*, *BLVRA* and *B*, *CYP7A1*, *HNF1a* and *4a*, *HPD*, *PKLR* and *SLC2A2 (GLUT2)* were observed.

From these target genes we selected six—*BLVRA*, *BLVRB*, *HPD*, *ABCC6*, *PKLR* and *APOA1*—for our further experiments. The latter two genes are part of the 451, which have an HNF4α binding site overlapping with the TSS (**S1 Fig**). Pyruvate kinase (PKLR) plays an important role in regulating glucose metabolism [7]. The other genes we have selected are also implicated in metabolism. 4-Hydroxyphenyl pyruvate dioxygenase (HPD) is an enzyme participating in tyrosine metabolism [32]. Mutations of the gene lead to a benign Mendelian disorder called Tyrosinaemia (type III). Apolipoprotein A1 (APOA1) plays primary role in lipid transport [33]. The biliverdin reductase genes catalyze the synthesis of bilirubin from biliverdin and thus participate in heme metabolism and the antioxidant pathway. Although the molecular function of ABCC6 is still unclear, it is protective against ectopic calcification, moreover, it might play a role in ATP homeostasis and in ROS elimination [23, 24, 34].

Our ChIP-qPCR experiments confirmed our initial hypothesis suggesting that ERK1/2 has both post-translational and probably transcriptional effects on HNF4α. These experiments showed that already short activation of the ERK1/2 pathway decreased the DNA binding capacity of HNF4α in living cells suggesting a post-translational effect. This rapid decrease became even more pronounced after 24h ERK1/2 activation suggesting the inhibition of *HNF4a* gene expression, as described earlier [29]. We chose the ChIP-qPCR method to detect the binding capacity alterations of the TF to a limited number of genomic loci to allow us reliable quantification of the changes. By using this approach, we could also perform several independent experiments, which was essential since the variability of ChIP experiment results is generally high.

Our experiments also proved that the rapid post-translational effect of ERK1/2 activation on HNF4α is mediated by the phosphorylation of the protein. We have shown that an *in vitro* translated HNF4α protein is phosphorylated at multiple sites by the activated ERK1. Several studies have revealed that HNF4α can be phosphorylated by several kinases, for instance, PKC, PKA, AMPK and p38. Phosphorylation can alter its DNA-binding affinity, intracellular localization and *trans*-activation capacity [35]. Phosphorylation of the highly conserved S87 by PKC drastically decreases the DNA binding capacity and stability of the protein [17]. Similarly, the cAMP-activated PKA phosphorylates HNF4α at the S142/S143 position [18]. This leads to reduced DNA binding activity of the TF, however, the reduced *trans*-activational potential could not be confirmed in luciferase reporter gene assay setup [18]. More recently, it has been shown that Thyroid-stimulating hormone (TSH) induces PKA, which leads to decreased nuclear localization of HNF4α in HepG2 cells [36]. AMPK has been defined as a metabolic master switch [8]. It phosphorylates the conserved S313 residue of HNF4α leading to the inhibition of the dimerization of the TF [20] and thereby its *trans*-activational capacity. AMPK activation dramatically decreases the transcription of various HNF4α target genes in hepatocytes. Finally, the role of p38 seems to be controversial. Some papers indicate that phosphorylation of HNF4α at residue T166/S167 by this kinase increases *trans*-activational potential of the TF [19, 37]. Others show indirect effect [20, 38], while some suggest an inhibitory role for p38 [9].

We have also investigated the functional relevance of the different residues shown to be phosphorylated by ERK1. In these experiments we used the luciferase reporter gene cloned downstream of the *ABCC6* promoter, a construct used in our previous studies [22, 24]. We have shown that this promoter is induced by HNF4α. In our present experiments we co-expressed this reporter gene with wild-type (wt) or different phosphomimetic HNF4α mutants.

The positive control mutant mimicking PKC phosphorylation resulted in significantly reduced reporter gene activity relative to the wt, as described earlier [17]. We have also analyzed the sites firstly identified in the present study (S451, T457/T459) and the sites corresponding to the previously described potential p38 site (T166/S167) [19]. In contrast to the previous studies, the phosphomimetic mutants of this site did not affect the *trans*-activational potential of HNF4α. In these experiments, only the site previously shown as targeted by AMPK (S313) showed diminished reporter gene activity [20, 38]. Collectively, our results clearly demonstrate that ERK1/2 activation results in HNF4α phosphorylation and reduced DNA binding capacity.

The ERK1/2 pathway is activated under several physiologic and pathologic conditions underlying the importance of our findings. For example, bile salts function as signalling molecules through the Sphingosine-1-phosphate receptor 2 (S1PR2) G protein coupled receptors (GPCRs), which activate ERK1/2 to control hepatic glucose, lipid and drug metabolism ([39–41]; [42] and references therein). The resulting rapid downregulation of HNF4α activity reduces the expression of the gluconeogenic genes *PEPCK* and *G-6-Pase* [40], or the gene encoding the major enzyme in bile acid synthesis, *CYP7A1* [43].

In addition to the GPCR pathway, bile acids also activate specific nuclear receptors (e.g. FXR and vitamin D receptor (VDR)). While VDR is able to directly activate ERK1/2 [44], FXR is the major bile acid-responsive ligand-activated transcription factor and it is responsible for bile acid homeostasis [45, 46]. FXR induces the expression of SHP (small heterodimer partner), an orphan nuclear receptor without DNA binding domain. SHP binds HNF4α and inhibits its binding to the target *cis*-regulatory elements (e.g. the bile acid response element (BARE) of the *Cholesterol 7α hydroxylase* (*CYP7A1*) gene promoter) [47]. SHP is also activated by ERK1/2 leading to an intricate network of HNF4α inhibition both in the short and longer term [42, 46, 48].

Finally, the ERK1/2 pathway is also activated by other mechanisms including oxidative stress (ROS) [49], growth hormones (such as HGF, EGF and FGF15/19 [24, 50]) and cytokines (IL1 and TNFα [9, 14, 19]), as well. These factors also lead to the reduced activity of HNF4α and thereby the downregulation of several genes. According to our results, the mechanism of the short-term inhibition of HNF4α is its phosphorylation, suggesting that ERK1/2 plays a pivotal role in the coordinated regulation of a great number of hepatic genes *via* the rapid post-translational modification of HNF4α.

## Materials and methods

### Cell culture

HepG2 human hepatoma cell line was obtained from ATCC (ATCC HB-8065) and cultured in Advanced MEM (ThermoFisher) supplemented with 10% FBS, 2mM L-glutamine, 100 U/ml penicillin and 100 mg/ml streptomycin. HeLa cells were obtained from ATCC and cultured according to the manufacturer’s instructions (DMEM supplemented with 10% FBS, 2mM L-glutamine, 100 U/ml penicillin and 100 mg/ml streptomycin). For EGF treatment, cells were changed to serum free medium 24h before the addition of the chemical, and then treated for 30 minutes or 24 hours with human recombinant epidermal growth factor (Sigma–Aldrich) at 100 ng/ml final concentration.

### *In vitro* phosphorylation assay

The reaction was performed in a mixture (30 μl final volume) containing kinase buffer [28], 500 ng ERK1 kinase (Sigma ERK1 kinase datasheet, cat number: E7407) HNF4α human recombinant protein with GST-tag at N-terminal (Abnova HNF4 alpha datasheet, cat number: H00003172-P01) and 20 uM ATP including 1 μCi [γ-^32^P] ATP. The reaction was initiated by the addition of ATP. After incubation at 30°C for 30 min, the reaction was stopped by adding 10 μl of 4X concentrated SDS sample buffer. Samples were subjected to SDS-PAGE using 10% running gels. After drying, gels were subjected to autoradiography for 2–12 hours.

### Phosphopetide mapping of HNF4α

The gel band containing HNF4α protein was processed, reductively alkylated with DTT and iodoacetamide and then digested with trypsin in 20 mM ammonium bicarbonate buffer. An aliquot was then run a Thermo/Dionex Ultimate RSLC nano system using a 75um x 15 cm C 18 PepMap column (Thermo/Dionex) coupled to a Thermo LTQ Orbitrap Velos Pro. We used a TOP 15 MS method (65 min linear gradient from 5–40% B (80% acetonitrile in 0.1% formic acid) with multi-stage activation (to detect neutral loss of phosphate from phosphopeptides). The obtained data file was analysed by the Mascot search engine against the sequence provided (HNF4α with N-terminal GST-tag). The phosphopeptide assignment was done on peptides above a mascot ion score of 20 which may include the same peptide a number of times as the system has detected that peptide on more than one occasion. The indication is that the higher the ion score for the peptide the more likely that the assignment is correct.

### HNF4α mutations

Wild-type plasmid containing the full human HNF4α gene and pcDNA5-FRT/TO expression backbone was purchased from Addgene. Amino acid numbering in this article refer to mutations of the human HNF4α gene, whereas mutations of the rat HNF4α gene are adjusted to the human numbering. Mutations were designed for the following serine or threonine phosphorylation sites creating phosphomimetic (glutamate or aspartate) or neutral (alanine) mutants: S87D, T166A/S167D, S313D, S451E, T451A/T459E. If two phosphorylation sites were adjacent or in very close proximity, both were mutated. Gene synthesis and site-directed mutagenesis were performed by the biotechnology company GenScript. The gene was re-cloned from pcDNA3+ into pcDNA5-FRT/TO plasmid.

### Transfection and luciferase experiments

HeLa cells were plated onto 96-well plates starting with 10.000 cells/well. FuGENE HD transfection reagent (Promega) complex containing serum-free medium and 2 μg total plasmid DNA was added to cells in growth medium. Triple co-transfection was performed with the phACCC6(-332/+72)Luc construct ((see [24]) composed of the *ABCC6* promoter fragment (-332/+72) cloned upstream of the luciferase coding cassette in the pGL3-Basic vector (Promega)), pcDNA5-FRT/TO plasmid encoding HNF4α variants (GenScript) and pRL-TK Renilla luciferase Control Reporter Vector (Promega). Cells were harvested and lysed after 48 hours. Luciferase activity was determined by Victor luminometric plate reader (Perkin Elmer) using the DualGlo Luciferase system (Roche). The obtained results were normalized firstly for the background noise, then for transfection efficiency by the co-transfected control reporter vector.

### ChIP (chromatin immunoprecipitation) assay

Formaldehyde was added directly to the culture media of flasks containing 10x10^6^ HepG2 cells, to a final concentration of 1%. After 10 min incubation at room temperature, fixation was quenched by adding 125 mM ice-cold glycine, and flasks were washed three times with ice-cold phosphate buffered saline (PBS). Cells were then scraped and washed three more times with PBS, each washing step being followed by sedimentation at 1,300 x g. Pellets were resuspended in 1mL lysis buffer (5 mM PIPES-pH = 8.0, 85 mM KCl, 0,5% NP-40, cOmplete Mini cocktail tablets (Roche) and incubated at 4°C for 15 mins with vortexing. Lysed cells were sedimented at 13,000 x g, resuspended in 500 μL of sonication buffer (1% SDS, 10 mM EDTA, 50 mM Tris-HCl-pH = 8.0, cOmplete Mini cocktail tablets) and sonicated on ice with an MSE sonicator (6 pulses of 15s each at 15% amplitude with 30s off between each pulse). The generated fragments were approximately 500 bp long, as determined experimentally. After sedimentation at 13,000 x g, the supernatant was transferred into a new tube, diluted ten times with IP buffer (0,01% SDS, 1.1% Triton X-100, 1.2 mM EDTA, 16.7 mM Tris-HCl (pH = 8.0), 167 mM NaCl) and supplemented with cOmplete Mini cocktail tablets. For each 500 μL extract, a mixture of 60 μL of Dynabeads protein A and 60 μL of Dynabeads protein G (ThermoFisher), conjugated with 2 μg anti-HNF4α mouse monoclonal antibody (Abcam ab41898) or anti-H3K27ac rabbit polyclonal antibody (Abcam ab4729) was added. After incubation at 4°C overnight, the beads were subsequently washed with buffer A (0.1% SDS, 1% Triton X-100, 2 mM EDTA, 20 mM Tris-HCl (pH = 8.0), 0.15 M NaCl), buffer B (0.1% SDS, 1% Triton X-100, 2 mM EDTA, 20 mM Tris-HCl (pH = 8.0), 0.5 M NaCl) and buffer C (0,25 M LiCl, 1% NP40, 1% Na-deoxycholate, 1 mM EDTA, Tris-HCl (pH = 8.0), at 4°C with permanent rotation for 5 mins. Following two washes with TE buffer (10 mM Tris-HCl, 10 mM EDTA, pH = 8.0), samples were eluted in 200 μL freshly prepared NaHCO_3_ (0,1 mM) supplemented with 1% SDS. Cross-links were reversed by overnight incubation at 65°C. Samples were incubated with 20μg/mL RNAse at 37°C for 1 hour, then proteinase K was added to 20 μg/mL and the mixture was incubated for 2 hours at 45°C. DNA was purified using High pure PCR template preparation kit (Roche). ChIP-seq libraries were prepared from 5 ng DNA with Ovation Ultralow Library Systems (Nugen) according to manufacturer’s instructions. Libraries were sequenced on Illumina HiScanSQ sequencer.

### ChIP-seq data processing

Raw sequence files of the ChIP-seq samples were processed using a computational pipeline [51] with the hg19 reference genome. ChIP-seq peaks were predicted using HOMER [52]. Artifacts, based on the blacklisted genomic regions of the Encyclopedia of DNA Elements [53], were removed from the peak sets using BEDTools [54]. RPKM (Reads Per Kilobase per Million mapped reads) values for the HNF4α sample were calculated on the summit -/+50 bp region of the peaks; for the H3K27ac sample were calculated on the whole region of the histone signal. Motif enrichment analysis was carried out by *findMotifsGenome*.*pl*. Pathway analysis was done by *annotatePeaks*.*pl* using the KEGG database [55]. The average read density was determined by *annotatePeaks*.*pl* [52]. Read distribution and average density heat maps were displayed by Java TreeView [56]. Histogram and box plot were performed using GraphPad Prism version 6.00 for Windows (GraphPad Software, La Jolla California USA).

### Quantitative PCR

A fraction of the DNA was used as a template for quantitative PCRs. The primers used were designed with BiSearch [57] and their sequences are shown in **Table 3**. PCR products were between 100 and 200 bp. qPCRs were performed in a total volume of 20 μL containing 1x SYBR green mix (Roche), a 1/10 fraction of ChIP-enriched DNA, and 250 nM primers in a 96-well plate. Using sonicated genomic DNA samples at different dilutions, we generated standard curves, and relative amounts of immunoprecipitated DNA were calculated by extrapolating from the dilution curves. All standards and samples were run in duplicate. Plates were read with a LightCycler 480 real-time PCR machine (Roche). Enrichment of a particular DNA fragment was calculated by comparing its relative concentration to control regions where no binding to the protein of interest is expected.

**Table 3:** List of qPCR primers.

Primer name	Primer sequence
ABCC6 promoter F	AGCCCATTGCATAATCTTCTAAGT
ABCC6 promoter R	ATGGAGACCGCGTCACAG
ABCC6 exon31 F	AAGTACACACAGCATGGCAG
ABCC6 exon31 R	AGGACCTAGCAATACACAGG
β-globin F	AGGACAGGTACGGCTGTCATC
β-globin R	TTTATGCCCAGCCCTGGCTC
APOA1 F	ATTGCAGCCAGGTGAGGAGAA
APOA1 R	TTAGAGACTGCGAGAAGGAG
BLVRA F	TTGTTTTGGAATGGGGGTGG
BLVRA R	AAAAGGGAAGGCTGTGGCAA
BLVRB F	CACCCTTTACCCTCTTTACC
BLVRB R	GCCTGTGCTTTTGTGTTTAC
HPD F	GATAGGGAAAACAGCCACCA
HPD R	TTGGATGATGAGGACACAGG
PKLR F	GTGGCTTACATGCTGTGGCT
PKLR R	TAGGTGGGTTTTGGAGAGGA

### Accession number

Sequencing data were submitted to NCBI’s Sequence Read Archive (SRA) database under accession number SRP096703 at the following URL:

https://www.ncbi.nlm.nih.gov/sra/?term=SRP096703

## Supporting information

S1 Fig Recruitment of HNF4α and H3K27ac at promoters. Histogram shows the average tag density of HNF4α and H3K27ac peaks at those 451 HNF4α binding sites which overlap with the transcription start site (TSS).
(TIF)

S2 Fig ChIP-qPCR results showing HNF4α occupancy on seven genomic regions of hepatic HNF4α target genes. Immunoprecipitation of chromatin from HepG2 cells was performed with anti- HNF4α or IgG antibody untreated or treated with EGF for 30 mins or 24 hours. Enrichment was compared to negative control region (Beta globin) for TF occupancy (y axis). HNF4α binding (black columns) is decreased after short EGF treatment (dark grey), which is further diminished after long EGF treatment (light grey). BLVR A: Biliverdin A, BLVR B: Biliverdin B, HPD: 4-hydroxyphenylpyruvate dioxygenase, PKLR: Pyruvate kinase, liver and RBC, ABCC6: ATP-binding cassette subfamily C, member 6 and APOA1: Apolipoprotein A1. S.D. is indicated on the figure.
(TIFF)

S1 Table HNF4α target genes identified in ChIP-seq experiment on vehicle treated HepG2 cells. (XLSX)

S2 Table P values of one-sample t-test on individual HNF4α target genes investigated by ChIP-qPCR. (XLSX)
