# Supplementary figures and images for: The transcriptional activity of hepatocyte nuclear factor 4 alpha is inhibited via phosphorylation by ERK1/2
Source: PLoS One. 2017 Feb 14;12(2):e0172020. doi: 10.1371/journal.pone.0172020 (PMC5308853; doi:10.1371/journal.pone.0172020)

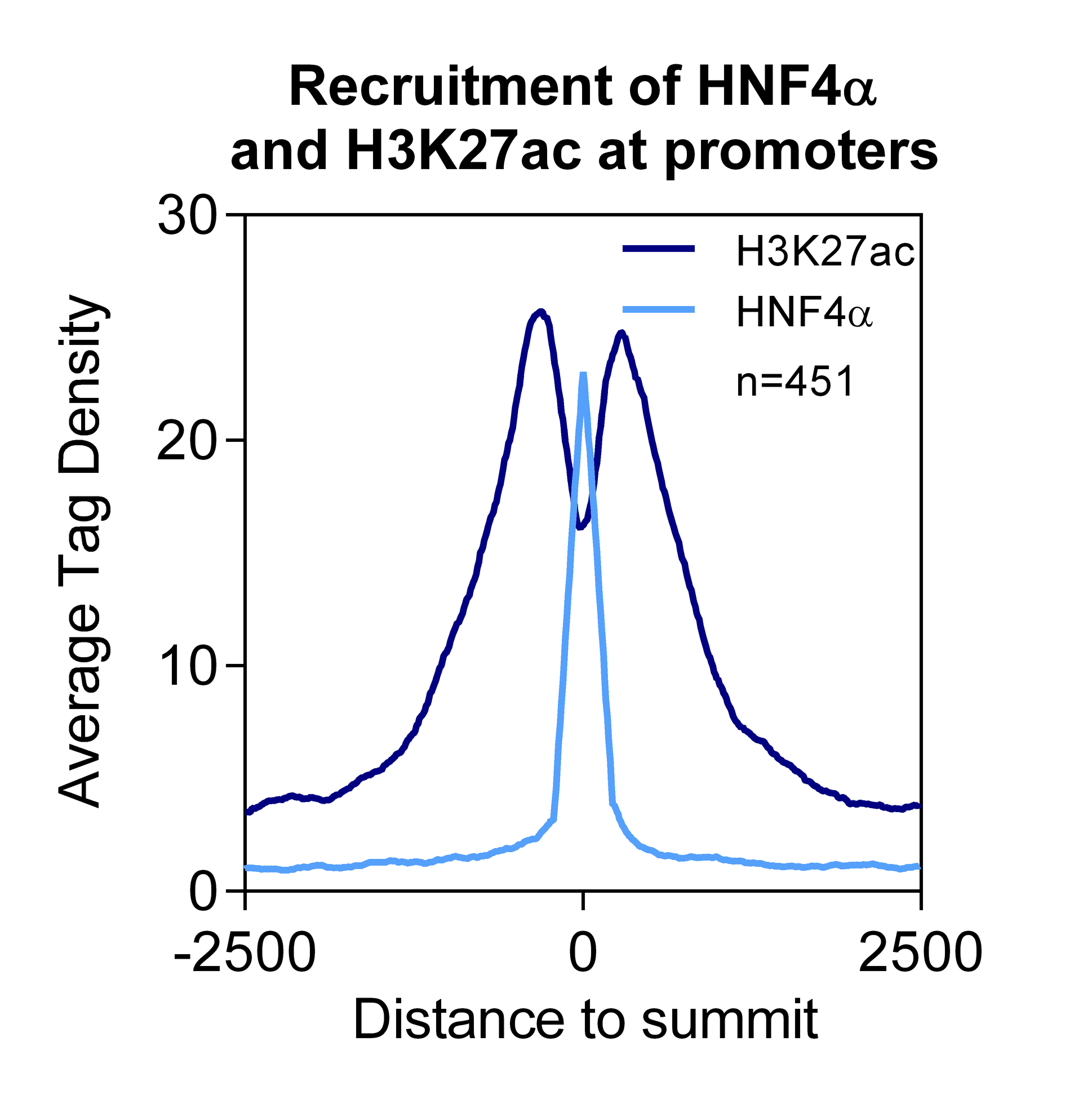

Supplement: S1 Fig — Histogram shows the average tag density of HNF4α and H3K27ac peaks at those 451 HNF4α binding sites which overlap with the transcription start site (TSS). (TIF) [file pone.0172020.s001.tif]

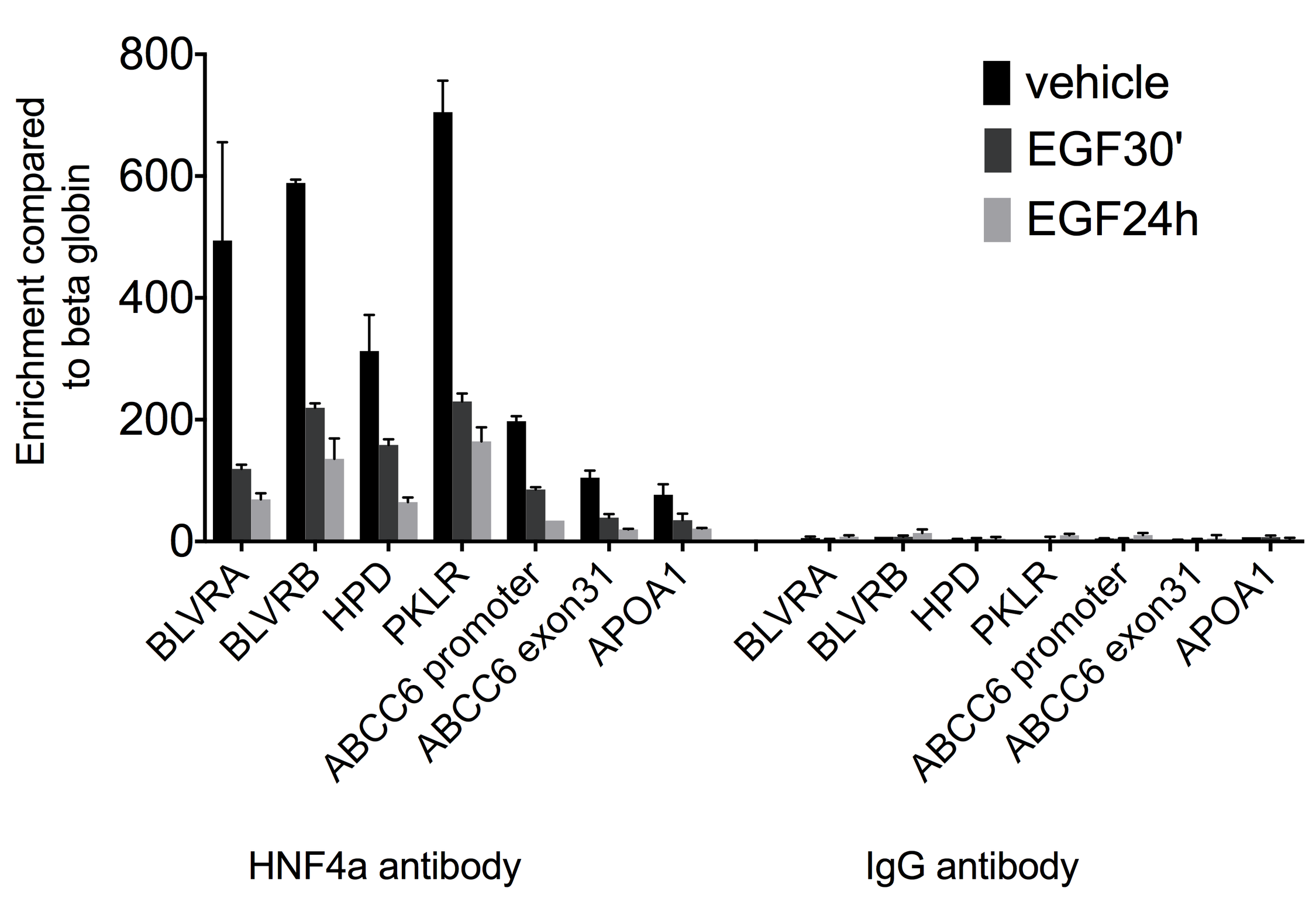

Supplement: S2 Fig — Immunoprecipitation of chromatin from HepG2 cells was performed with anti- HNF4α or IgG antibody untreated or treated with EGF for 30 mins or 24 hours. Enrichment was compared to negative control region (Beta globin) for TF occupancy (y axis). HNF4α binding (black columns) is decreased after short EGF treatment (dark grey), which is further diminished after long EGF treatment (light grey). BLVR A: Biliverdin A, BLVR B: Biliverdin B, HPD: 4-hydroxyphenylpyruvate dioxygenase, PKLR: Pyruvate kinase, liver and RBC, ABCC6: ATP-binding cassette subfamily C, member 6 and APOA1: Apolipoprotein A1. S.D. is indicated on the figure. (TIFF) [file pone.0172020.s002.tiff]
